# Enhancement of ROS Production by Catechin Is a Primary Effect of Increased Azole Efficacy in *Nakaseomyces glabratus* (*Candida glabrata*) Cells Lacking the *ERG6* Gene

**DOI:** 10.3390/pathogens13100834

**Published:** 2024-09-26

**Authors:** Nora Tóth Hervay, Daniel Eliaš, Lucia Černáková, Juraj Jacko, Marcela Habová, Natália Chovancová, Yvetta Gbelská

**Affiliations:** 1 Department of Microbiology and Virology, Faculty of Natural Sciences, Comenius University in Bratislava, Ilkovicova 6, 842 15 Bratislava, Slovakiayvetta.gbelska@uniba.sk (Y.G.); 2 Department of Nuclear Physics and Biophysics, Faculty of Mathematics, Physics and Informatics, Comenius University in Bratislava, Mlynska Dolina, 842 48 Bratislava, Slovakia; jacko@fmph.uniba.sk

**Keywords:** *Nakaseomyces glabratus*, *Candida glabrata*, *ERG6*, catechin, OSR

## Abstract

Fungal infections have become an important public health problem. Currently, there are only three available classes of antifungals for the treatment of invasive infections. Two of them, azoles and polyenes, target the synthesis of ergosterol or bind to sterols. A promising strategy to improve current therapies is the use of natural compounds in combinational therapies with the existing antifungals. In this work, we analyzed the changes in the susceptibility of the mutant strain of *Nakaseomyces glabratus* (*Candida glabrata*) lacking the *ERG6* gene (encoding the sterol C-24 methyltransferase in ergosterol biosynthesis) in the presence of catechin and antifungal azoles. The reduced content of ergosterol in the *Cgerg6*Δ mutant resulted in the increased tolerance of the mutant cells to both azoles and polyenes. The combination of catechin with fluconazole or miconazole led to the growth inhibition of the azole-resistant *Cgerg6*Δ mutant strain. In the presence of catechin and miconazole, the *Cgerg6*Δ mutant fails to properly activate the expression of genes encoding the transcription factors *Cg*Yap1p and *Cg*Msn4p, as well as the gene expression of *CgCTA1*, which are involved in oxidative stress response and lead to the intracellular accumulation of ROS. Finally, we show that catechin administration reduces mortality in a *Galleria mellonella* model infected with *C. glabrata*. Our work thus supports the use of catechin in combination therapies for fungal infections and shows that the *CgERG6* gene could be a potential new drug target.

## 1. Introduction

Yeast belonging to *Candida* spp. are ubiquitous human commensals; however, in an immunocompromised host, they cause serious infections [1]. While *Candida albicans* remains the main cause of systemic candidiasis, in recent years, there has been a shift toward non-albicans *Candida* species as causes of infection, such as *C. glabrata* (recently reclassified as *Nakaseomyces glabratus*), *C. parapsilosis*, *C. tropicalis*, *C. krusei*, or *C. auris* [2,3]. Invasive candidiasis is a life-threatening fungal infection with a high mortality rate in patients suffering from immunologic deficiencies or undergoing surgical interventions and transplantations [4,5]. The mortality rate of invasive candidiasis varies depending on the *Candida* species but reaches approximately 40–60% [6,7]. An important prerequisite for the acquisition of virulence traits in *C. glabrata* is the ability to adapt and be resistant to environmental variations, which allows the pathogen to colonize many different niches and organs [8,9]. Compared to other *Candida* species, *C. glabrata* is intrinsically less susceptible to fluconazole, the prime antifungal drug used in the treatment of infections; rapidly develops resistance to antifungal drugs; and has a well-defined oxidative stress response (OSR)—including both enzymatic and non-enzymatic mechanisms—that appears to play a central role in the survival of *C. glabrata* in macrophages [10,11].

Unfortunately, as only a few types of antifungal agents, such as polyenes, azoles, echinocandins, and a novel triterpenoid, are available, the therapy choices for fungal diseases are highly restricted [12,13,14]. Strategies for the development of novel antifungal agents against fungal infections include novel antifungals, antifungal chemosensitizers, drug repurposing, and new targets. One way to extend the lifespan of antifungals is the use of plant-derived compounds which can enhance the activity of the existing drugs and reduce the rate at which the pathogens gain resistance [15]. In recent decades, interest in natural polyphenols as chemosensitizers has increased [16,17,18]. Species of the *Camellia* genus have been highlighted as possessing antimicrobial activity and as a great source of polyphenols, such as catechins [19,20,21,22]. Several reports point to the increased efficacy of antifungal azoles and polyenes when combined with catechins, with a strong effect also against antifungal-resistant yeast isolates and biofilms [16,17,23,24,25]. The studies also indicate that catechin exhibits both antioxidant and prooxidant effects at concentrations commonly used in in vitro models (50–250 µM). The antioxidant effects are caused by the effective scavenging of superoxide anions by catechin. The prooxidant effects are caused by the increased generation of catechin-derived oxidants related to their metal-chelating ability, reducing properties, and radical scavenging ability [26,27,28,29].

Another possibility to solve the problem of antifungal drug resistance is to find novel drug targets. Triazole antifungals act as inhibitors of ergosterol biosynthesis and are the frontline therapy for fungal infections. Due to the uniqueness and essentiality of ergosterol for fungal organisms, the disruption of ergosterol homeostasis is considered as a promising strategy for novel antifungal development. Navarro-Martinez et al. [23] showed that epigallocatechin gallate can indirectly influence the ergosterol biosynthesis via folate cycle inhibition. The inhibition of ergosterol biosynthesis is the result of the decreased cellular pool of S-adenosylmethionine, which serves as a donor of methyl group for the sterol C-24 methyl transferase, encoded by the *ERG6* gene. Erg6p is one of the three specific enzymes, including Erg4p and Erg5p, which are not present in humans. The loss-of-function mutations of Erg6p in yeast lead to altered drug susceptibility and defective phenotypes related to membrane integrity and permeability [30]. Therefore, it is hypothesized that the product of the *ERG6* gene could serve as a potential new drug target [31,32].

In our previous study, we have shown that catechin potentiates the activity of miconazole in *C. glabrata* laboratory strain and *C. glabrata* clinical isolates [33]. In this work, we investigated the effect of catechin in the *C. glabrata* mutant with a deleted *CgERG6* gene. The absence of *Cg*Erg6p leads to the increased resistance of the mutant cells not only to polyenes, but also to antifungal azoles.

## 2. Materials and Methods

### 2.1. Yeast Strains, Oligonucleotides, and Media

*Candida glabrata* strains used in this study were *Cglig4*Δ *lig4::HIS3trp1* [34] (in further text designated as wt) and its isogenic derivative *Cglig4*Δ *erg6*Δ*lig4::HIS3erg6::TRP1* [35] (appointed as *Cgerg6*Δ). *Cglig4*Δ deletion strain was kindly provided by Patrick van Dijck (KU Leuven, Belgium). The *C. glabrata* cells were cultivated in a YPD medium (1% yeast extract, 2% peptone, 2% glucose). For solid media, 2 g per 100 mL of agar was added to the liquid medium mentioned. The oligonucleotides used in this study are listed in Table S1.

### 2.2. Susceptibility Assays

The susceptibility of *C. glabrata* strains to various drugs was tested by spot assays. Overnight, yeast cultures were grown in the YPD medium, diluted to a density of 1 × 10^7^ cells/mL. An amount of 5 µL aliquots of tenfold serial dilutions (10^7^, 10^6^, and 10^5^ cells/mL) of overnight cultures were spotted onto YPD plates and incubated at 30 °C for 2 days. The experiments were repeated five times.

### 2.3. Isolation of RNA and Quantitative PCR

The relative levels of gene expressions were assessed by quantitative PCR (qPCR). Yeast cells were grown in the YPD medium to the mid-logarithmic phase. *C. glabrata erg6*Δ cells were further cultivated in YPD broth with/without miconazole (0.5 μg/mL), catechin-hydrate (2 mg/mL), or their combinations for 2 h. The isolation of RNA was performed using the GeneJET RNA Purification Kit (Thermo Scientific, Waltham, MA, USA) with the following modification: 200 μL of yeast lysis buffer was added to re-suspend the pellet, followed by a 60 min incubation at 30 °C. All further steps were conducted according to the manufacturer’s protocol. An amount of 1 µg of RNA was used as a template for first-strand cDNA synthesis using Revert AidTM H Minus MMuLV Reverse Transcriptase (Thermo Fischer Scientific, Waltham, MA, USA). qPCR was prepared using the HOT FIREPol^®^ EvaGreen^®^ qPCR Mix Plus (ROX), 5× (Solis Biodyne, EU, Tartu, Estonia). Amplification was carried out in the 7900 HT Fast Real-Time PCR System (Applied Biosystems, Waltham, MA, USA). The melting curves obtained after the completion of the qPCR cycles were used to verify the presence of specific amplicons. The reporter signals were analyzed using the ABI SDS 2.2.2 software (Applied Biosystems, Waltham, MA, USA). *CgACT1* was used as a reference gene. The gene expression levels were determined according to Livak and Schmittgen [36]. The gene transcript levels in the control samples were set as 1. Any expression value ≥ 2 was considered as up-regulation, and expression value ≤ 0.5 as down-regulation. The results are expressed as the mean values of three independent experiments (± standard deviation). For statistical analyses, the Kruskal–Wallis test was used. A *p* < 0.05 (*) was considered statistically significant.

### 2.4. Detection of Intracellular ROS Levels

The production of ROS was measured using dihydrofluorescein diacetate (H_2_DCFDA) which produces fluorescence after being attacked by ROS. The cells were grown to a late exponential phase in YPD. A total of 1 × 10^9^ cells in 10 mL of YPD were pretreated with catechin-hydrate (2 mg/mL), miconazole (0.5 μg/mL), or fluconazole (25 μg/mL), or with combinations of the chemicals for 2 h at 30 °C. The cells were washed in phosphate-buffered saline (PBS). A suspension of 1 × 10^5^ cells was prepared in PBS and incubated with 25 μM H_2_DCFDA (SigmaAldrich, St. Louis, MO, USA, dissolved in DMSO) in a 96-well plate. The DCF fluorescence signal was measured using GloMax Discover Microplate Reader (Promega Corp., Madison, WI, USA) at 0, 30, 60, and 90 min at excitation and emission wavelengths of 475 and 500–550 nm, respectively. The time–response curves were compared by first-order linear regression using Graphpad Prism.

### 2.5. Fluorescence Anisotropy Measurements

The cells grown in the YPD medium at 30 °C to the mid-logarithmic phase were washed twice in Tris–Cl buffer (10 mmol/L, pH 7.0). The cells (OD600 of 0.1) were labeled with DPH or TMA-DPH, in the final concentration of 1.5 × 10^−7^ mol/L. Plasma membrane fluidity was determined using the Luminescence Spectrometer Perkin Elmer LS 55 with L-format measurement (Perkin Elmer, Waltham, MA, USA). The excitation wavelength was 360 nm; the emission wavelength was 430 nm. Anisotropy (rs) was calculated as described by Bencova et al. [37]. For statistical analyses, the one-way analysis of variance (ANOVA) and post hoc Dunnett multiple comparisons with control were used (unpaired *t*-test).

### 2.6. Galleria Mellonella Infection

The antifungal effectivity of catechin-hydrate in vivo was evaluated by using a *Galleria mellonella* model. Overnight, *C. glabrata* cultures were washed twice with PBS (pH 7.2) and adjusted to 5 × 10^8^ cells/mL. Three independent groups of 10 randomly chosen larvae were infected with 10 μL of yeast cultures via the last left proleg with a precision syringe (Hamilton microliter syringes, cemented-in needle, Type 701N cap. 10 μL). The treated groups (n = 30) were also injected with 10 μL of catechin-hydrate (diluted to 2 mg/mL in PBS) to the last right proleg. PBS-injected larvae were used as negative control groups to monitor the trauma of injection (n = 30). The larvae were incubated at 30 °C for 7 days. The survival of the larvae was monitored every 24 h by visual inspection. Melanized and non-motile larvae were considered as dead and were subtracted from the experiment. The difference between the groups was compared using the long-rank test.

## 3. Results

### 3.1. Catechin Enhances the Antifungal Activity of Azoles in C. glabrata

**Figure 1:**
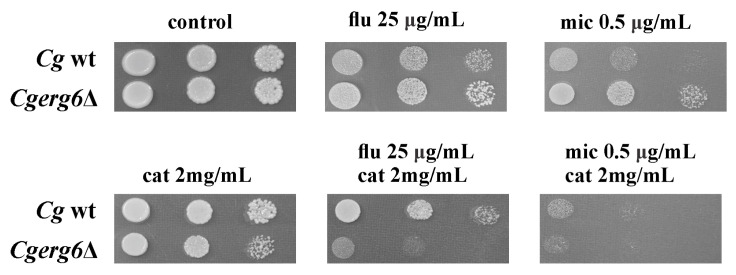
Susceptibility of *C. glabrata* wt strain and the *C. glabrata erg6*Δ mutant to fluconazole and miconazole alone and in combination with catechin. An amount of 5 µL aliquots of tenfold serial dilutions (10^7^, 10^6^, and 10^5^ cells/mL) of overnight cultures were spotted onto YPD plates and incubated at 30 °C for 2 days.

Based on the results obtained by evaluating the effect of catechin in combination with antifungal azoles in *C. glabrata* laboratory strain as well as *C. glabrata* clinical isolates [33], in this work, we explore the ability of catechin to alter the resistance profile of the *C. glabrata erg6* mutant to antifungal azoles. To explore whether catechin altered the antifungal susceptibility of the *C. glabrata erg6*Δ mutant, we performed spot assays. Figure 1 shows that the growth of the *C. glabrata* strain in the YPD medium was not affected by the presence of catechin alone. However, the co-administration of catechin and fluconazole or miconazole resulted in the increased susceptibility of the *C. glabrata erg6*Δ mutant strain. The combined use of miconazole and catechin leads to the growth inhibition of both *C. glabrata* strains; however, the effect was more pronounced in the mutant strain (Figure 1). An almost complete absence of colony development was evident at a miconazole concentration of 0.5 µg/mL in the *C. glabrata erg6*Δ mutant.

### 3.2. Increased Intracellular ROS Generation Induced by Catechin and Azoles in C. glabrata

**Figure 2:**
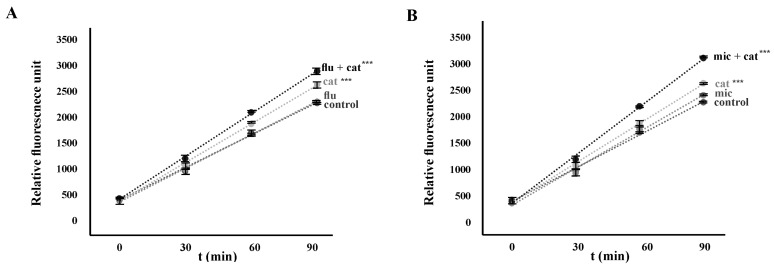
Production of ROS by *C. glabrata* erg6Δ deletion mutant cells in the presence of 25 μg/mL fluconazole (flu), 0.5 μg/mL miconazole (mic), 2 mg/mL catechin (cat), or their combinations. The time points represent the average of three independent experiments (± standard deviation). The points were fitted with a line. The differences between the slope of the control and catechin, and fluconazole with catechin are extremely significant *p* < 0.001 (***) (**A**). The differences between the slope of the control and catechin, and miconazole with catechin are extremely significant *p* < 0.001 (***) (**B**).

According to previous reports [26,38], catechins are considered “double-edged swords” in cells, as they can exhibit prooxidant activity in addition to their antioxidant properties. To verify whether susceptibility profiles could be influenced by the prooxidant activity of catechin, we examined the intracellular concentration of ROS in *C. glabrata erg6*Δ cells. The presence of intracellular ROS during catechin exposure was assessed using the fluorescent probe H_2_DCFDA-2′,7′-dichlorodihydrofluorescein diacetate. In the presence of catechin, a significant increase in the intracellular concentration of ROS was observed in the cells of the mutant *C. glabrata erg6*Δ (Figure 2A,B). An even greater increase in intracellular ROS concentration was observed in the presence of both the antifungal azole (fluconazole or miconazole) and catechin (Figure 2A,B), indicating that the induction of ROS production by the simultaneous presence of catechin and azole in the *C. glabrata erg6*Δ mutant cells was additive.

### 3.3. Expression of the Genes Involved in OSR in C. glabrata

**Figure 3:**
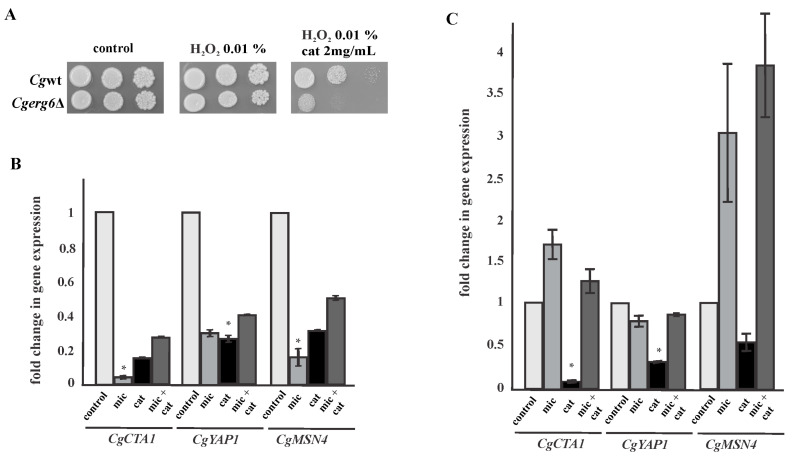
Growth of parental strain (wt) and *Cgerg6*Δ mutant in the presence of hydrogen peroxide and catechin. An amount of 5 μL aliquots of tenfold serial dilutions (10^7^, 10^6^, and 10^5^ cells/mL) of overnight cultures were spotted onto YPD plates and incubated at 30 °C for 2 days (**A**). The relative gene expression levels of *CgCTA1*, *CgYAP1*, and *CgMSN4* in *C. glabrata erg6*Δ mutant (**B**). The relative gene expression levels of *CgCTA1*, *CgYAP1*, and *CgMSN4* in *C. glabrata* wild-type (**C**). The results are expressed as the mean values of three independent experiments (± standard deviation) normalized to the *CgACT1* gene expression level. The graph shows the relative changes in gene expression after 2 h of incubation in the presence of miconazole (mic), catechin (cat), or their combination (mic + cat) compared to untreated samples set as 1. A *p* < 0.05 (*) was considered statistically significant.

The prooxidant effect of catechin was further confirmed by spot tests (Figure 3A) in the presence of the oxidative stress inducer hydrogen peroxide. The susceptibility phenotype of *C. glabrata erg6*Δ mutant cells increased significantly in the simultaneous presence of catechin and hydrogen peroxide (Figure 3A). *C. glabrata* has a well-defined oxidative stress response that includes both enzymatic and nonenzymatic mechanisms controlled by the well-conserved transcription factors Yap1, Skn7, Msn2, and Msn4. The strong resistance to oxidative stress in *C. glabrata* is believed to be mediated through the functions of a single catalase (*Cg*Cta1p), two superoxide dismutases (*Cg*Sod1p and *Cg*Sod2p), and the glutathione and thioredoxine detoxification systems [39,40]. In the next experiment, we quantified the transcript levels of a single *CgCTA1* gene encoding catalase and the genes encoding the transcription factors *CgYAP1* and *CgMSN4* in cells incubated with miconazole or catechin alone or in their simultaneous presence by RT-PCR. As shown in Figure 3B, in the *C. glabrata* mutant lacking Erg6p, hydrogen peroxide inducible expression of the catalase-encoding gene *CgCTA1* was abolished both in the presence of miconazole and catechin alone as well as in the simultaneous presence of both compounds. In the wild-type strain, significantly decreased expression of the gene *CgCTA1* occurred only in the presence of catechin, which may be due to the decreased expression of the gene *CgYAP1* in the presence of catechin (Figure 3C). The reduced expression of the *CgMSN4* gene was also observed in the presence of catechin, while the presence of miconazole induced the expression of this gene encoding transcription factor. The transcript levels of the genes encoding the transcription factors *Cg*Yap1p and *Cg*Msn4p were also reduced in the *C. glabrata erg6*Δ mutant cells (Figure 3B). Based on these results, we propose that a dysfunction of the antioxidant system occurred in the *C. glabrata erg6*Δ mutant cells in the presence of catechin and azoles.

### 3.4. Plasma Membrane Fluidity

To investigate whether catechin can alter the plasma membrane fluidity of the *C. glabrata erg6*Δ mutant strain, we measured the fluorescence anisotropy of whole cells using TMA-DPH and DPH probes. We did not observe any statistically significant effect of catechin or its combination with miconazole on plasma membrane fluidity in the *C. glabrata erg6*Δ mutant cells (Table 1).

### 3.5. Catechin Reduces the Mortality of Infected Wax Moth Model

**Figure 4:**
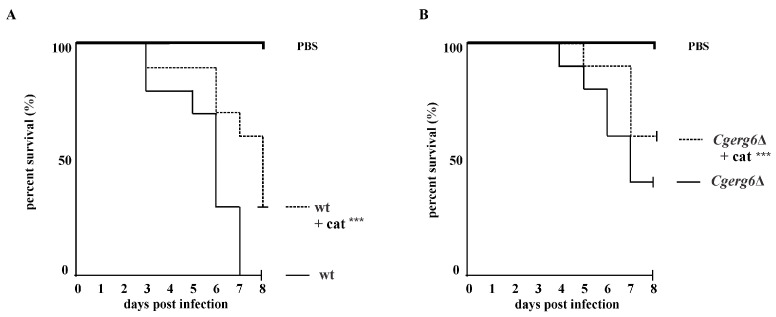
Effect of catechin (cat; 2 mg/mL) on the survival of *G. mellonella* larvae after inoculation with *C. glabrata* wt (**A**) and *Cgerg6*Δ mutant cells (**B**). The difference between the treated and non-treated groups is extremely significant with *p* < 0.001 (***).

Our in vitro results demonstrate that the *CgERG6* gene is not required for the viability of *C. glabrata*. To determine the effects of *CgERG6* gene deletion in vivo, we compared the survival rates of a *Galleria mellonella* model for fungal infection, after infection with the *C. glabrata* wild-type strain and the *C. glabrata erg6*Δ mutant strain. We also assessed the viability of *G. mellonella* larvae infected with *C. glabrata* strains injected together with catechin. The larvae of *G. mellonella* experienced 100% mortality by day 7 post-inoculation with the *C. glabrata* wild-type strain (Figure 4A). As Figure 4B shows, the absence of sterol C-24 methyltransferase leads to the reduced virulence of the *C. glabrata erg6*Δ mutant, and the catechin administration further reduced the fungal burden of the *C. glabrata erg6*Δ mutant cells. Although the absence of *Cg*Erg6p reduced the mortality of the *G. mellonella* larvae, we did not achieve their 100% survival. The results of the statistical long-rank test used show statistically significant differences between the groups.

## 4. Discussion

Due to the increasing prevalence of life-threatening fungal infections and the ability of human pathogenic yeast to develop resistance to the current treatment options, there is a great need to find and develop new strategies to combat them. One way to prolong the utility of antifungal drugs, and combat drug-resistant pathogens, is the administration of molecules that restore fungal susceptibility to the approved drugs. Several studies have reported that plant-derived natural polyphenolic compounds—catechins—can enhance the antifungal azole effectivity in various *Candida* yeast species [17,24]. Navarro-Martinez et al. [23] showed that the inhibition of folic acid biosynthesis by catechin affects ergosterol biosynthesis through negative feedback on Erg6p (sterol C-24 methyltransferase). In this work, we have analyzed the ability of catechin to modulate the *C. glabrata erg6*Δ mutant azole susceptibility. We show that the administration of catechin, together with azoles fluconazole or miconazole, augments the growth inhibition of the *C. glabrata erg6*Δ mutant. However, the observed increased susceptibility of the *C. glabrata* mutant, devoid of Erg6p, to antifungal azoles in the presence of catechin suggests that Erg6p is not the direct target of catechin.

The combination of catechin with antifungal azole leads to increased ROS production in the *C. glabrata erg6*Δ mutant. This observation corroborates the results of da Silva et al. [41], who showed that the combination of flavonoids with fluconazole induces apoptotic death in *C. tropicalis*, where the generation and intracellular accumulation of ROS seem to play a crucial role. Based on the increased susceptibility of the *C. glabrata erg6*Δ mutant to hydrogen peroxide, observed in the presence of catechin, we propose that catechin induces ROS production in the cells, which is responsible for the enhanced activity of antifungal azoles in the presence of catechin. However, the reduced content of ergosterol in the *C. glabrata erg6*Δ mutant could contribute to the increased susceptibility of mutant cells to hydrogen peroxide. Dupont et al. [42] point to the antioxidant role of ergosterol in a computational study based on quantum chemistry. Intracellular antioxidant enzymes play a crucial role in protecting cells from oxidative stress-induced damage [41,43]. The absence of *Cg*Erg6p leads to the down-regulation of the *CgCTA1* gene encoding the single catalase in *C. glabrata* cells, as well as the decreased expression of the genes *CgYAP1* and *CgMSN4* encoding the transcription factors that play a pivotal role in oxidative stress tolerance. Therefore, we hypothesize that the *C. glabrata erg6*Δ mutant cells are unable to effectively repair the oxidative damage induced by the presence of catechin. In addition to ROS-related mechanisms, catechin–protein interaction is believed to be involved in the mechanisms by which catechins exert their biological activities. The phenolic structures of catechins have the potential to interact strongly with proteins due to the interaction of their hydrophobic benzene rings with the proline residues of proteins and the hydrogen bonding potential of phenolic hydroxyl groups [44]. The structural/conformational properties of hydrogen bonds have also been proposed as mechanisms for catechin interactions with transcriptional factors [41]. Therefore, we cannot exclude the possibility that a dysfunction in the antioxidant system occurs in cells in the presence of catechin. The up-regulation of *CgMSN4* in the wild-type strain, and the down-regulation of this gene in the *C. glabrata erg6*Δ deletion mutant in the presence of miconazole indicate that Msn4p is crucial for resistance to azole in *C. glabrata*. *C. glabrata* can rapidly adapt to environmental changes with Msn2/Msn4p functioning independently, which is the opposite of what is observed in *Saccharomyces cerevisiae* [44].

Previous studies have shown that catechins interact with biological membranes by adsorption or penetration into the lipid bilayers and affect the fluidity and permeability of the membrane [45,46,47,48]. The fluidity of the plasma membrane determines the passive permeability of the membrane, the extent to which various molecules such as drugs, products of metabolism, and toxins can enter or leave the cell unaided by dedicated transport proteins [49]. Frallicciardi et al. [50] showed that sterols modulate the solute permeability by affecting the membrane phase state. In contrast to the isogenic *C. glabrata* wild-type strain [33], we did not observe any statistically significant effect of catechin or its combination with miconazole on the plasma membrane fluidity in the *C. glabrata* Δ mutant. The increased stiffness and organization of the plasma membrane in the *C. glabrata erg6*Δ mutant due to zymosterol accumulation, resulting from the absence of sterol C-24 methyl transferase [35], could prevent its fluidization by catechin. Based on these observations, we propose that the interaction of catechin with the *C. glabrata* plasma membrane is dependent on its sterol profile.

Finally, we assessed the potential of catechin in the treatment of *Candida* infections in the in vivo invertebrate model *Galleria mellonella*. *C. neoformans erg6*Δ mutant and mutants with reduced *ERG6* expression in *C. albicans* have been reported to have significantly reduced virulence in *G. mellonella* infection models [31,51]. Catechin injection of the *G. mellonella* larvae infected with the *C. glabrata erg6*Δ mutant resulted in a reduction in larvae death. The survival of the larvae infected with the *C. glabrata erg6*Δ mutant was even better, both with or without the catechin injection, compared with the isogenic wild-type strain. Thus, increased ROS production by catechin may play an important role in the survival of the *G. mellonella* larvae infected with the *C. glabrata erg6*Δ mutant.

## 5. Conclusions

Taken together, we showed that catechin sensitizes *C. glabrata* cells lacking the *CgERG6* gene through the induction of ROS production. Combined therapy targeting specific fungal enzymes allows the use of lower doses of antimycotics and may consequently lead to the decreased production of resistant isolates in *Candida* yeast species. Our results show that catechin could be used together with conventional antifungal azoles for the treatment of fungal infections, allowing the use of lower doses of antimycotics.

## Figures and Tables

**Table 1:** The influence of miconazole and catechin on the values of fluorescence anisotropy in the *C. glabrata erg6*Δ mutant cells.

	Control	Mic	Cat	Mic + Cat
Fluorescent Probe				
DPH	0.254 ± 0.006	0.250 ± 0.010	0.251 ± 0.015	0.246 ± 0.010
TMA-DPH	0.323 ± 0.009	0.323 ± 0.011	0.318 ± 0.009	0.322 ± 0.009

## Data Availability

Data generated or analyzed during this study are available in the published article.

## Supplementary Materials

The following supporting information can be downloaded at https://www.mdpi.com/article/10.3390/pathogens13100834/s1, Table S1: List of oligonucleotides.
